# Therapeutic Vascular Compliance Change May Cause Significant Variation in Coronary Perfusion: A Numerical Study

**DOI:** 10.1155/2012/791686

**Published:** 2012-03-05

**Authors:** S. Nobari, R. Mongrain, E. Gaillard, R. Leask, R. Cartier

**Affiliations:** ^1^Department of Biomedical Engineering, McGill University, Montreal, QC, Canada H3A 2B4; ^2^Department of Cardiovascular Surgery, Montreal Heart Institute, Montreal, QC, Canada H1T 1C8; ^3^Department of Mechanical Engineering, McGill University, Montreal, QC, Canada H3A 0C3; ^4^Department of Chemical Engineering, McGill University, Montreal, QC, Canada H3A 2B2

## Abstract

In some pathological conditions like aortic stiffening and calcific aortic stenosis (CAS), the microstructure of the aortic root and the aortic valve leaflets are altered in response to stress resulting in changes in tissue thickness, stiffness, or both. This aortic stiffening and CAS are thought to affect coronary blood flow. The goal of the present paper was to include the flow in the coronary ostia in the previous fluid structure interaction model we have developed and to analyze the effect of diseased tissues (aortic root stiffening and CAS) on coronary perfusion. Results revealed a significant impact on the coronary perfusion due to a moderate increase in the aortic wall stiffness and CAS (increase of the aortic valve leaflets thickness). A marked drop of coronary peak velocity occurred when the values of leaflet thickness and aortic wall stiffness were above a certain threshold, corresponding to a threefold of their normal value. Consequently, mild and prophylactic treatments such as smoking cessation, exercise, or diet, which have been proven to increase the aortic compliance, may significantly improve the coronary perfusion.

## 1. Introduction

In some pathological conditions like aortic stiffening and calcific aortic stenosis, the microstructure of the aortic root and the aortic valve leaflets is altered in response to stress resulting in changes in tissue thickness, stiffness, or both [1, 2].

Aortic stiffening is the consequence of changes in the properties of the arterial wall that accompany aging and arterial hypertension. Increased aortic stiffness is associated with an elevation of systolic blood pressure, a reduction in diastolic blood pressure, and thus a widened pulse pressure [3]. This widened pulse pressure leads to left ventricular hypertrophy and increases the risk of stroke and myocardial infarction [4]. Accelerated arterial stiffness has been linked to diabetes [5, 6], hyperglycemia, hyperinsulinemia, and impaired glucose tolerance [7, 8].

Aging of the aortic valve (AV) is characterized by cuspal thickening [9] and loss of extensibility (i.e., increase of stiffness) [10], which can lead to progressive changes in AV function. The most common disease of the AV is calcific aortic stenosis (CAS), found in 2% of patients over 65 years old and in 4% of those over 85 [11]. This pathology is characterized by the occurrence of calcified nodules on the valve leaflets, which can grow over time, thickening and stiffening the leaflets and eventually critically interfering with valve opening and potentially closing [12]. Currently, the most common treatment for CAS is the calcified valve replacement by a mechanical or a bioprosthetic valve [13]. CAS is the leading single etiology of valve disease necessitating valve replacement, accounting for a major fraction of the approximately 300,000 valve replacement surgeries worldwide each year [14].

Aortic stiffening and CAS are thought to affect coronary blood flow [15, 16]. Reduced aortic distensibility (i.e., increased aortic stiffness) results in a decrease of diastolic backflow that aids coronary perfusion, disturbing thus the oxygen demand/supply balance of the myocardium [17]. The presence of a severe CAS in patients markedly reduced the coronary flow reserve (CFR), a well-known characteristic of the distensibility of the coronary arteries [16]. This reduction of CFR can be explained by the concomitance of reduced myocardial supply as a result of decreased coronary perfusion pressure, and increased myocardial metabolic demand as a result of increased left ventricular workload.

 In recent years, we have begun to address the critical area of fluid-structure interaction (FSI) and have developed our model of aortic valve including Valsalva sinuses but without coronary arteries [18]. Several other numerical studies have been carried out to improve the quality of the surgical procedures but without considering the coronary structures [19–22]. The goal of the present study was to include the flow in the coronary ostia in the previous FSI model, to analyze the effect of diseased tissues (aortic root stiffening and CAS) on coronary perfusion, and to link prophylactic treatments as smoking cessation, exercise, or diet with coronary perfusion changes. Inclusion of the coronaries will allow us to obtain hemodynamic variables related to coronary flow such as perfusion level and velocity distribution. It would also allow investigating the possible mutual effect between coronary pathologies, and aortic wall stiffening and valve dynamics. However, this bidirectionality is out of the scope of this paper since it would require the global structure of the coronary vessels. Therefore, the current paper will present the one-way interaction between aortic wall stiffening and CAS, and their effect on coronary perfusion. Even though coronary autoregulation—the intrinsic ability to maintain a constant blood flow despite changes in perfusion pressure—might mitigate some of this interaction [15, 23, 24], in a first approach, in order to simplify the analysis, autoregulation was not incorporated into the model.

## 2. Methods

The finite element software LS-DYNA (Livermore Software Technology Corporation, Livermore, CA, USA) was used to perform all FSI simulations.

### 2.1. Model Geometry

**Figure 1:**
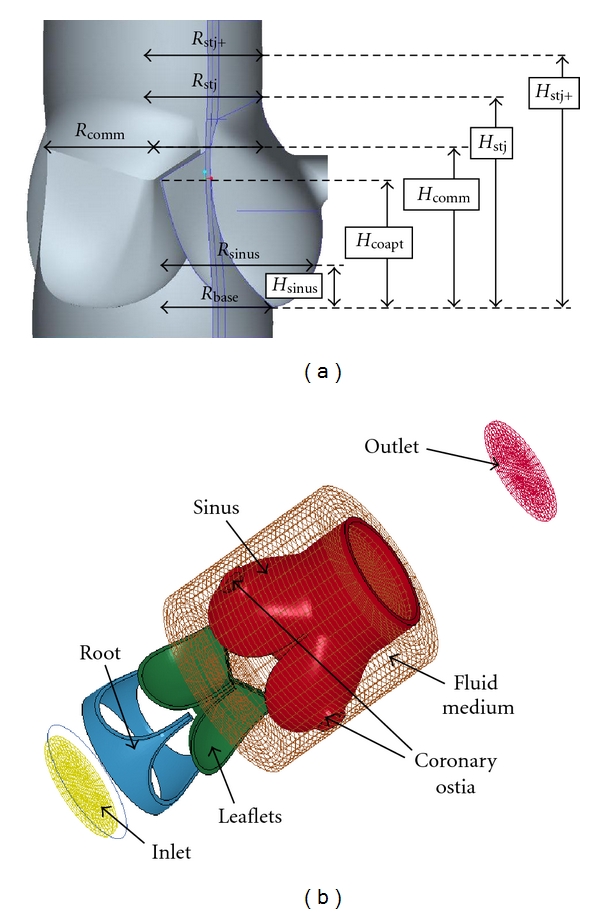
(a) Anatomical parameters used to create the 3D geometric model of the aortic root. (b) Exploded view of the final 3D CAD model.

A generic, anatomically inspired 3D model of the aortic valve was derived from a combination of imaging modalities (MRI, 3D-digitization) and pathological data as previously described by Ranga et al. [18] and Campbell et al. [25]. Dimensions for the anatomical parameters were taken as the average of various values reported in previous studies [26]. A drawing showing these parameters is presented in Figure 1(a), while Figure 1(b) shows a finalized version of the model in an exploded view along the axial direction. The model is made of two main domains, the solid and the fluid, each containing subassemblies. Three components comprise the solid medium: the aortic root, the leaflets, and the sinuses. The sinus component additionally includes a portion of the ascending aorta and the two coronary ostia. The fluid medium also consists of three components: an inlet, an outlet, and the central region. This region embeds the entire cardiac tissue to accommodate for the fluid-structure interaction. The fluid external to the aortic wall represents the pericardial fluid and is considered to be stagnant and at atmospheric. The base of the valve and inlet of the reservoir are connected together restricting the displacement in the axial direction. This guarantees that the two domains remain connected during the analysis.

The model was meshed in ANSYS, discretizing the solid components into 10,892 shell elements. These shell elements were used instead of solid brick elements because they can have several integration points allowing bending to be modeled while satisfying the physics of the model. The fluid medium, on the other hand, consisted of 24,500 hexahedral elements. This was found to be an adequate mesh density after doing mesh refinements and mesh independency tests by increasing node numbers up to twice their initial value. After convergence, the percent differences in velocity results using a point-to-point Euclidean norm were less than 2.78%. This meshing was then transformed into the format of an input file including the whole geometry, material properties, boundary conditions, and loads to be analyzed in LS-DYNA.

### 2.2. Material Properties

 Cardiac tissue is a highly complex material. In the context of large strains, heart tissues can be modeled using nonlinear material properties [22, 27–29]. However, under normal physiological conditions, the strain in the aortic root varies in the range of about 10% [30, 31]. A recent study has shown that even though the stress-strain curve of the cardiac tissue is nonlinear, it can be subdivided into two linear regions: one at low strain range (below 15%) and another at high strain rates [32]. At low strain rates, this linearity is even more pronounced and a study by our group has shown that at these physiological strains the stress-strain curve of aortic tissue can essentially be considered linear [33]. Given the related advantages, we implemented a linear elastic material property to model the healthy cardiac tissue with a Young's modulus (incremental modulus) of 3.34 and 4.00 MPa for the aortic root and leaflets, respectively, and a Poisson's ratio of 0.45. These values are in the physiological range and are similar to the values used in previous studies [22, 34]. In fact, it has been shown that with this assumption the stress levels are within acceptable ranges as compared to hyperelastic modeling [35, 36]. The main reason for neglecting the viscous effects was to isolate the effect of the elastic component in the artery. This allowed us to investigate the consequence of aortic wall stiffening and valve thickening on the energy stored in this elastic component, which would ultimately affect coronary perfusion. The roll of viscous terms become significant when the smooth muscle cells are activated [37, 38]. Since the microstructure of the vessel has not been the focus of this study and the smooth muscle cells have not been considered, the viscous effects could be neglected.

As for blood, its density was set to 1060 Kg/m^3^. At high levels of shear rate (>50 s^−1^), the dynamic viscosity of blood varies between 3 and 4 mPa.s, meaning that in general a simple Newtonian relationship may be used in studies of the larger vessels [39]. In summary, the fluid was considered Newtonian with a density of 1060 kg · m^−3^ and a dynamic viscosity of 3.5 mPa.s. 

### 2.3. Boundary Conditions

#### 2.3.1. The Fluid Medium

 There are four sets of boundary conditions in the fluid medium that need to be defined. These boundary conditions are imposed at the aortic root, the ascending aorta, and the two coronary ostia. In order to account for the pulsatility of the flow, the inlet condition at the aortic root is taken to be the difference between the ventricular pressure and the aortic pressure. Therefore, the outlet of the ascending aorta has a free boundary condition, meaning fluid can flow freely, an approach borrowed from previous studies [27]. The result is that the entire system is under an overall smaller pressure and the shell elements of the solid medium are subjected to a reduced transvalvular load while maintaining the pressure gradient across the valves.

 As for the two coronary ostia, it is known that the hydraulic resistance of the coronary vascular bed is largely time dependent and that much of this phenomenon is attributed to the vasocontraction of the coronary vessels [40]. This resistance is one of the controlling factors of how much flow will enter each of the coronaries. In order to incorporate this into the model and study the effect of pathologies on coronary perfusion, two types of boundary conditions were imposed at the ostia depending if we were studying a healthy or pathological case. First, in the healthy case, clinical values of coronary flow [41] were imposed as boundary conditions. This leaves the pressure and velocity profile unconstrained and allows them to self-establish. Second, for pathological conditions, this same pressure obtained at the ostia for the healthy case is used as the new boundary condition, which will allow the flow to self-establish. This enables us to study the effect of a single parameter on the coronary perfusion. In previous studies by our group, zero coronary flow conditions were investigated and leaflet dynamics and stresses were compared to clinical data [18]. A comparison of the results from this previous study, a hypertensive case, and the current model with clinical data are presented in Section 3.1.1 and commented further in the discussion section.

#### 2.3.2. The Solid Medium

 In the solid medium, boundary conditions were applied at the aortic ring, the ascending aorta, the coronary ostia, and the leaflets. These boundary conditions constrain the rigid body motion (twisting, rotation, translation) but are not so limiting as to overrestrict the dynamics of the structure allowing for deformation [42]. To achieve this end, the geometry of the model contains a significant portion of the ascending aorta to fully constrain the outlet ring without affecting valvular dynamics. This constrain restricts motion and rotation in any direction at the top edge of the ascending aorta. On the other hand, at the level of the inlet ring, a constraint was applied to prevent the model from deforming and movement in the axial direction only. Hence the radial motion, and thus root expansion, was not constrained at the inlet.

The contact between leaflets was defined using CONTACT_AUTOMATIC_GENERAL in LS-DYNA environment, which is a single surface contact and checks for penetration along the entire length of the free (unshared) shell edges. This is a critical point in the modeling procedure since it prevents the shell elements from penetrating through each other and creating unrealistic leaflet motion. Finally, the coronary ostia were fully constrained in all rotational and translational degrees of freedom to avoid element deformation, which could possibly lead to coronary closure.

### 2.4. Governing Equations

The simulation of the complete cardiac cycle is performed using a fluid-structure interaction approach. Additionally, for the fluid flow part, the blood velocity is calculated in the context of the ALE formulation of the Navier-Stokes equations, which can be obtained by replacing the convective velocity in the standard Navier-Stokes equations by the relative velocity to the moving mesh. The general form is as follows:

(1) $$\begin{array}{c} \rho \frac{\partial \nu }{\partial t}+\rho \left(\nu -w\right)\cdot \nabla \nu -\nabla \cdot \sigma =0\;\;\text{in}\;\Omega_{f},\\ \nabla \cdot \nu =0\;\;\text{in}\;\Omega_{f}, \end{array}$$

where *ρ* is the fluid density, *v* the velocity vector of the fluid in a fixed coordinate system, *t* is the time, *w* the velocity of the fluid medium, and *σ* the Cauchy stress tensor defined by:

(2) σ=−pI+τ,

where *p* denotes the fluid pressure and *τ* the viscosity stress tensor.

The fluid-structure problem presented here consists of a fluid medium *Ω*_*f*_ and an immersed solid medium *Ω*_*s*_. In order to capture the fluid-structure interaction, these two mediums need to be coupled. This coupling is obtained by applying a no slip condition

(3) $$\nu_{f}-\nu_{s}=0,$$

where *ν*_*f*_ and *ν*_*s*_ represent the fluid and the structure velocity, respectively, in the coupling interface.

## 3. Results

### 3.1. The Solid Medium

 The engineering parameters of interest in the solid medium include the leaflet morphologies, leaflet velocities, and leaflet stresses. Although the solid medium contains the aortic root, sinuses, and ascending aorta, the most accurate clinical data available are related to the leaflets.

#### 3.1.1. Leaflet Morphologies and Dynamics

**Figure 2:**
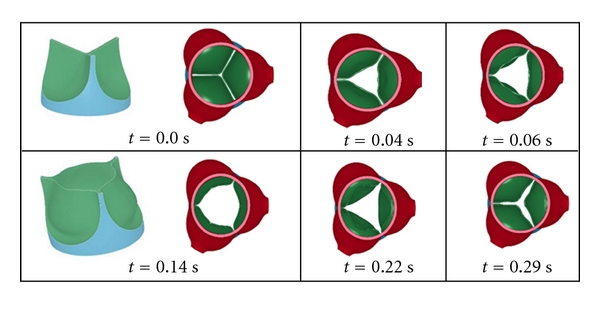
The computed opening and closing patterns of the leaflets, seen from the aorta. Note that at time *t* = 0.0 s, the leaflets are in perfect contact. The apparent gap in the first frame corresponds to the shell (Phantom) thickness.

 Looking from the ascending aorta back towards the left ventricle, known as the short-axis view, reveals some key features. An important aspect that can be quantified clinically is the cross-sectional valve opening during the cardiac cycle. The computed leaflet morphologies during the opening and closing phases are presented in Figure 2.

For a more quantitative assessment, it is also possible to track the velocities at the leaflet tips. Some common aspects of the leaflet dynamic histories that have been clinically quantified by echocardiography studies are the rapid valve opening time (RVOT), rapid valve opening velocity (RVOV), rapid valve closing time (RVCT), rapid valve closing velocity (RVCV), and ejection time (ET). For each of these five parameters, average healthy values have been determined [43–46]. These values are compared to those derived from the current model in healthy (120/80 mmHg: Δ*P* = 40 mmHg) and hypertensive (140/90 mmHg: Δ*P* = 50 mmHg) conditions and are presented in Table 1.

In the FSI study by Ranga et al. in 2006 [18], zero coronary flow was considered, and the values for RVOT, RVCT, and ET were reported as 102.5, 85, and 280 ms, respectively.

#### 3.1.2. Leaflet Stresses

 In this section, both the principal and von-Mises stresses are reported and examined both quantitatively and qualitatively. The principal stresses in the leaflets were observed at two instants of the cardiac cycle, one at the beginning of systole and the other one at mid-systole (0.072 seconds into the cardiac cycle). In each of these moments, a preferential direction of these stresses was noticed in a circular arrangement around the attachment edge of the leaflet. Conversely, stress in the inner area, or belly, of the leaflet was more scattered, representing a less structured environment. This particular distribution was also qualitatively similar to previous reports and known stress patterns on the leaflets [38, 47].

**Figure 3:**
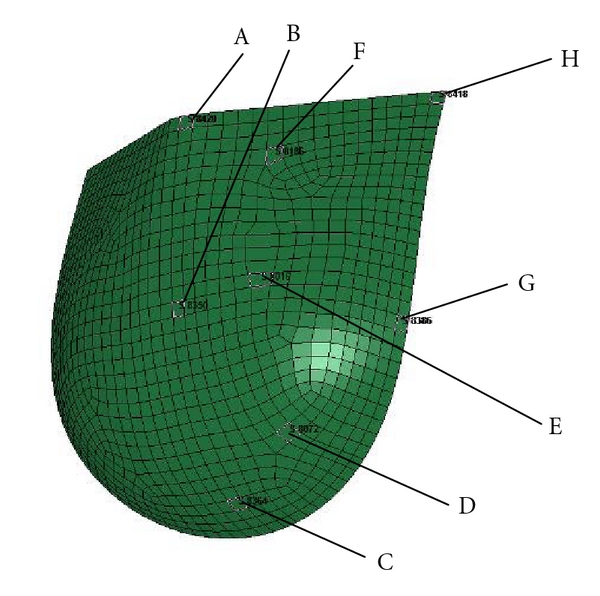
Illustration of the selected FE elements from which the von-Mises stresses are sampled.

 The stress levels were also assessed by comparing them to previously reported values under similar conditions. The locations of the elements sampled for comparison of their stress histories are given in Figure 3. These particular elements spatially match those from the previous study of the leaflet stresses during the dynamic actions of the valve by Gnyaneshwar et al. [48].

The specific values computed for the stress magnitudes at these locations for both models and their percent differences are presented in Table 2.

#### 3.1.3. The Fluid Medium

 Validation in the fluid medium can be accomplished to a certain degree by comparing bulk flow properties of the model to known physiological data and also by searching for known flow patterns such as diastolic recirculation regions, which are known to occur, but are more difficult to quantify.

 Blood velocities were sampled in the model at three locations: the flow through the valve at the level of the commissures and the sinotubular junction as well as flow through the coronary ostia. For the first two locations, comparisons to clinical data can be made over the entire cardiac cycle regarding both the total flow ejected and the temporal distribution of this flow. For the coronary flow, the shape of the waveform is less precisely known, although certain aspects, such as the total flow and the general shape, have been documented.

**Figure 4:**
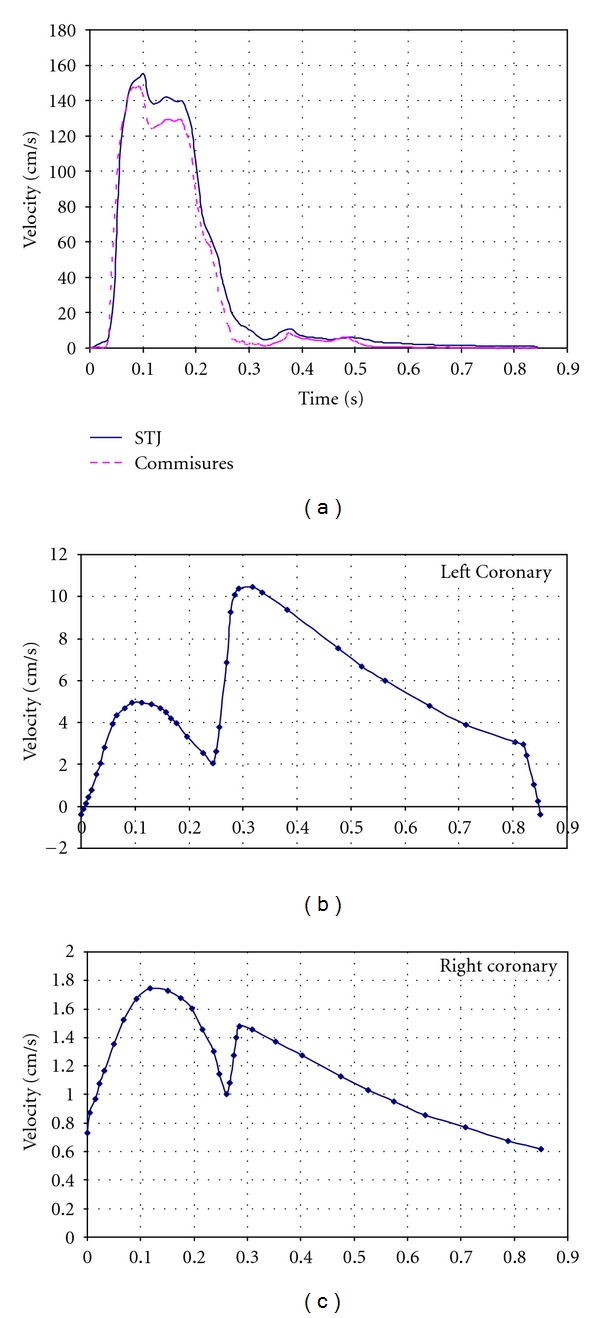
Computed velocity waveforms at (a) the STJ and commisures, (b) the left ostia and (c) the right ostia.

 The computed velocities at the STJ, commissure, left, and right ostia are provided in Figure 4 with a peak velocity of 1.41 m/s occurring at 0.087 s. The fluid exhibits a rapid acceleration followed by a deceleration slightly smaller in magnitude, which is known to be the case physiologically. Coronary perfusion data are close to the known physiological values with an overall coronary perfusion of 100 mL/min or approximately 4% of the cardiac output.

**Figure 5:**
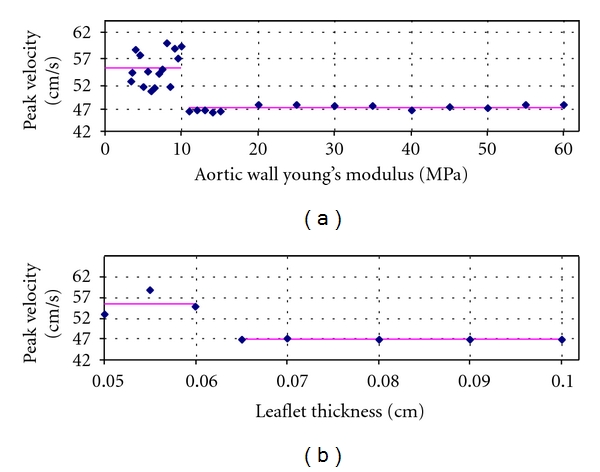
Behaviour of coronary peak velocity as a function of (a) aortic wall stiffness and (b) leaflet thickness.

We also considered the impact of varying the stiffness of the aortic wall elastic modulus from 3.34 MPa to 60 MPa and the thickness of the aortic valve leaflets from 0.05 to 0.1 cm to simulate aortic stiffening and CAS, respectively [49, 50]. In order to allow the coronary flow to self-establish, the prescribed flows at these locations were removed and the ostia were defined as outlets with no boundary conditions. Twenty-nine and eight different values were used for aortic wall stiffness and leaflet thickness, respectively.  Figure 5 represents the peak coronary velocity with respect to different aortic wall stiffness values ranging from 3.34 to 60 MPa and leaflet thicknesses from 0.05 to 0.1 cm.

As it can be seen from the figure above, the coronary flow exhibits slight variations up to certain critical values of stiffness and thickness for which a marked drop is observed. Although interesting, this phenomenon warrants to be confirmed with clinical evidence. 

## 4. Discussion

A model of the aortic valve including flow engaging in the coronary ostia was presented in this paper. An FSI analysis was performed using explicit LS-DYNA to study the aortic stiffening and the dynamics of the valve, and their effect on the coronary flow. The ALE method was used for the FSI analysis of this study. The results from this analysis in terms of leaflet morphologies, leaflet stresses, timing during the opening and closing phases, and velocities at critical points were presented. The leaflet morphologies during the opening and closing phases, shown in Figure 2, were comparable with previous studies and the valve opens to 69% of the cross-sectional area of the aortic ring [20]. Similar time-lapse images presented by other authors have demonstrated the same qualitative triangular orifice opening and leaflet billowing [19, 20, 22, 51].

For verification, known clinical parameters of rapid valve opening and closing time and velocities were presented in Table 1. We showed that our results match the echocardiography data reasonably well. The only exception would be RVCV, in which we reported a larger percent difference. We believe the reason for this occurring could be the smooth shape of the sinus structure that we synthetically generated with pathological data. Indeed, as it was demonstrated by Katayama et al. [52], the RVCV could be sensitive to the morphology of sinuses. We also reported that the results from a hypertensive case predict a more rapid opening and closing with a higher acceleration as expected. By comparing these results to those obtained by Ranga et al. [18] without coronary flow, we showed that the zero coronary flow tended to significantly increase the opening and closing times, while the results obtained in this study were closer to the clinical data obtained from echocardiography.

Leaflet stresses were presented in Section 3.1.2, and results from our simulation were compared to the study done by Gnyaneshwar et al. [48]. Similar points were selected as to match those in the reference study. For both studies the results of stress values range between 0-1 MPa. Additionally, the models are also in agreement as to the locations of the stress minima and maxima (Figure 3). Differences were calculated, these values ranged from 0.06 MPa to 0.15 MPa with an average difference of 0.078 MPa. 

Results obtained from the fluid domain including velocity at STJ or commisures were also in good agreement with previous studies. Another interesting result was obtained from a temporal examination of the velocity plots. Blood flow through commissures and STJ, in a sense, represent the bulk flow of the blood through the aortic valve. A peak velocity of 1.41 m/s occurred at 0.087 s, which is in agreement with previous studies that have reported peak velocities of 1.35 ± 0.35 m/s [35].

Results were also achieved for cases of aortic wall stiffening. These results revealed an impact of aortic wall stiffening on coronary flow. As shown in Figure 5, elastic modulus of the aortic wall was increased to a higher value than their healthy state, which caused a significant drop in coronary peak velocity after a threshold. This drop could be correlated to the decrease in coronary flow reserve (CFR) previously described in clinical studies [3, 17]. Aortic distensibility is a major determinant of left ventricular afterload [17]. Any deterioration of aortic distensibility (i.e., aortic stiffening) will result in ventricular-arterial mismatch and left ventricular dysfunction, which may prove critical in certain stages of disease. Aortic stiffening may lead to an early return of the reflected arterial pulse wave causing an increase of systolic blood pressure (SBP) and a decrease in diastolic blood pressure (DBP), thus increasing the pulse pressure. Greater SBP increases myocardial oxygen consumption, reduces left ventricular ejection fraction, and increases left ventricular afterload inducing left ventricular hypertrophy. Myocardial blood supply depends largely on pressure throughout diastole and the duration of diastole, so the decrease of DBP can compromise coronary perfusion resulting in subepicardial ischemia. Moreover, left ventricular hypertrophy also reduces coronary flow [17].

These results showed that below a value of aortic wall elastic modulus being equivalent to three times the normal value of aortic wall elastic modulus, the peak coronary velocity remained constant. This observation could be correlated to the beneficial effects of smoking cessation, exercise, and diet on arterial compliance. Oren et al. [53] revealed, in a clinical study in 2006, that smoking cessation for 6 months significantly improved arterial stiffness. Oscillatory compliance rose from 0.051 ± 0.023 to 0.063 ± 0.03 mL/mmHg, which corresponds to an increase of 23%. A short time of smoking cessation has a strong effect on the arterial compliance. Exercise plays an important role on arterial compliance recovery too. Tanaka et al. [54] determined the role of habitual exercise on age-related decrease in central arterial compliance. They demonstrated that arterial compliance fell from about 2 mm^2^/mmHg in sedentary healthy young subjects to values of about 1.2 to 1.3 mm^2^/mmHg in middle-aged and older sedentary humans. But in the highly trained middle-aged and older subjects (on a 3-month exercise period), exercise appeared to reduce the decline in compliance with aging by about 50%. Again a short time of regular exercise has a strong effect on the arterial compliance. Finally diet has also a strong effect on arterial compliance. Nestel et al. [55] studied the impact of eating fish and fish oil on systemic arterial compliance during 4 weeks in fifteen obese people. They showed that the systemic arterial compliance rose significantly of about 85%.

Consequently, the results we obtained associated with those of Oren et al. [53], Tanaka et al. [54], and Nestel et al. [55] showed that a short period of smoking cessation, regular exercise, or diet may restore the peak coronary velocity to a normal value and therefore reestablish a good coronary perfusion.

The increase of aortic valve leaflets thickness (mimicking CAS) led to a decrease in coronary peak velocity. Above a certain threshold, a marked drop in peak velocity occurred. These results were again in agreement with previous studies [16, 56]. In patients with CAS and a normal coronary angiogram, the CFR is significantly lower [50]. The CFR capacity of the coronary arteries depends on at least three main components: (1) micro- and macrovascular resistance, (2) myocardial resistance, and (3) the effect of viscosity [57]. In some pathologic conditions like aortic stiffening and CAS, changes in one or other of these factors may lead to an impairment of the CFR capacity.

It is known that aortic compliance is reduced as the cardiac tissue becomes aged and pathological [58]. Recently it has been shown that a stiff aorta is associated with a reduction in coronary blood flow [3, 56, 59]. However, there is little information relating coronary flow and aortic stiffness in humans [60]. An explanation for the drop in coronary flow observed in our simulation could be linked to the fact that 70–90% of the coronary perfusion occurs during diastole [61, 62] and also the fact that it is during this phase that blood is driven towards the coronary arteries by the elastic recoil of aorta (Windkessel model) [63]. This elastic recoil is the potential energy stored in the walls during systole and released during diastole to aid leaflet closure and blood perfusion through coronaries. Approximately 50% of the stroke volume is directly forwarded to the peripheral circulation [64]. Peripheral resistance and elastic extension of the aortic wall are responsible for storage of the other 50% of the stroke volume, the storage volume [65]. Reduction in aortic compliance will induce an increase on the impedance to the ventricular ejection which will lead to a decrease in coronary flow [66].

For a less compliant aortic wall, the impact of the reduction in elastic recoil would be direct as less energy is stored in the wall during systole. As for the case where the leaflets have thickened, this loss in the potential energy could be linked to the increase in blood velocity through the valves (due to reduction of effective orifice area) and the associated pressure drop. In that context, we believe that the hydraulic resistance and the vessel compliance could compound to produce the observed sudden drop in the perfusion.

Several assumptions and simplifications were made in the present study. A linear elastic model was used to mimic aortic root and aortic valve leaflets behavior, which leads to a short computing time compared to nonlinear models. However, in the context of aneurismal diseases, the linear elastic model used for its simplicity and ease of calculations would need to be expanded to nonlinear elastic in order to take into account for the large deformations. In addition, to investigate the aneurysm rupture process, it would be required to generalize the assumption of an isotropic model, which is sufficient for global assessment, to anisotropic model to integrate the vascular microstructures.

As for boundary conditions, the inlet boundary condition at the ventricular level is considered to be the pressure difference between the ventricle and aorta. This is an inevitable constraint imposed on the model to avoid introducing instabilities and extensive run-times in an already complex FSI model. This assumption will cause the aortic wall to experience a smaller pressure, specifically during mid-systole. As a result aortic root expansion will be reduced, which could affect the leaflet opening and closing pattern. The percent difference observed for RVCV could also be due to this reduced expansion and loss of distensibility in the sinuses. This assumption might cause the drop observed in the coronary perfusion to have a minor shift across the range of wall stiffness and leaflet thickness studied. Moreover, the leaflets thickness was considered constant on the whole leaflet surface, whereas this one varies along the leaflet. The leaflets are the thinnest in the belly, their load-bearing part. They become thicker towards the line of attachment and the leaflet free edge. The thickest part is the nodule of Arrantius, just below the center of the free edge [67]. Finally, the coronary ostia were only considered in this study and not the complete coronary arteries.

## 5. Conclusion

In this study, we have shown that a moderate increase in aortic stiffness and aortic leaflet thickness (CAS) will lead to a noticeable drop in coronary perfusion. Considering the fact that short-term smoking cessation, regular exercise or diet could cause a mild change in aortic compliance; this study suggests that such preventions and/or prophylactic treatments could significantly improve the coronary perfusion. The thresholding phenomena observed via a drop in the coronary flow, due to varying the stiffness and thickness of the aortic wall and leaflet tissues, are very interesting as it suggests a possible interaction between valvular and coronary diseases but warrants further analysis with exhaustive clinical data.

 The incorporation of the coronary ostia improved the opening and closing valve dynamics, compared to a model without the coronary arteries. The model is sensitive enough to differentiate between normal and slightly hypertensive conditions showing distinct impact on the valves dynamics.

The proposed model that incorporated perfusions in the coronary ostia has the potential to investigate the impact of flow disturbances in the aortic root on the coronary flow and eventually the impact of coronary obstructive diseases on aortic stiffening and aortic valve leaflets dynamics. It would also be possible to examine coronary perfusion variations due to mechanical and geometric modifications of the aortic root using clinical metrics for validation of the approach. Such investigations are not possible with previously described models of the aortic root without any coronary structures.

## Figures and Tables

**Table 1:** Comparison between FSI and echocardiography leaflet dynamics.

	Current model (healthy case)	Current model (Hypertensive case)	Echocardiography (Healthy)	% difference between the healthy case and echo
RVOT ms	53	44.6	46.0	14.14
RVOV cm/s	25.7	34.3	29.2	12.75
RVCT ms	52.7	40.1	47.0	11.43
RVCV cm/s	16.3	20.7	23.6	36.59
ET ms	276.0	248.2	329.0	17.52

(RVOT: rapid valve opening time, RVOV: rapid valve opening velocity, RVCT: rapid valve closing time, RVCV: rapid valve closing velocity, ET: ejection time) healthy case (120/80 mmHg: Δ*P* = 40 mmHg) and hypertensive case (140/90 mmHG: Δ*P* = 50 mmHg).

**Table 2:** Comparison of the maximum stress (MPa) during the cardiac cycle in selected locations represented in Figure 3 in the proposed model and Gnyaneshwar model.

Location	Proposed model	Gnyaneshwar	% difference
A	0.492	0.15	106.54
B	0.311	0.20	43.44
C	0.480	0.42	13.33
D	0.390	0.24	47.61
E	0.380	0.28	30.30
F	0.158	0.13	19.44
G	0.914	0.90	1.54
H	0.478	0.40	17.76
